# Xerostomia and Salivary Gland Hypofunction in Patients with Oral Lichen Planus Before and After Treatment with Topical Corticosteroids

**DOI:** 10.2174/1874210601711010155

**Published:** 2017-03-31

**Authors:** Hala Al-Janaby, Haytham El-Sakka, Manal Masood, Walimuni Ashani W. Mendis, Linda M. Slack-Smith, Richard Parsons, Agnieszka M. Frydrych

**Affiliations:** 1 School of Dentistry, The University of Western Australia, Perth, Western Australia; 2 School of Occupational Therapy and Social Work, Curtin University, Perth, Western Australia

**Keywords:** Dry mouth, Oral lichen planus, Salivary gland hypofunction, Topical corticosteroids, Xerostomia

## Abstract

**Background:**

Oral lichen planus and mouth dryness are common pathoses, yet not entirely understood. These two conditions may be associated, with a few studies investigating the relationship between mouth dryness and oral lichen planus providing conflicting results. None of the studies have explored the specific impact of disease treatment on mouth dryness.

**Objective:**

The purpose of this observational before and after comparison study was to examine the effect of treatment of oral lichen planus with topical corticosteroids on mouth dryness.

**Methods:**

Nineteen subjects with oral lichen planus were evaluated for the severity of xerostomia using a xerostomia inventory and a visual analogue scale. Stimulated and unstimulated whole salivary flow rates, unstimulated salivary pH and buffering capacity were also measured. All subjects were evaluated before and after treatment with topical corticosteroids.

**Results:**

All subjects reported xerostomia before treatment with topical corticosteroids, with 79% reporting a significant improvement (*P* = 0.03) after treatment. Topical corticosteroid treatment was not associated with statistically significant differences in stimulated or unstimulated salivary flow rates, unstimulated salivary pH or buffering capacity.

**Conclusion:**

The results of this study suggest that treatment of oral lichen planus with topical corticosteroids may decrease the severity of dry mouth symptoms.

## INTRODUCTION

Oral lichen planus (OLP) is a chronic, systemic, immunologically mediated disease, affecting about 0.1 - 2.0% of the general adult population [1-3]. It affects men and women of all ethnic groups, however, women are more commonly affected and there is a higher prevalence in the Indian population [1]. The disease can occur at any age with the typical age at presentation ranging from 30-60 years [1]. The pathogenesis of this disease is not completely understood, although a cell-mediated immune response appears to play a major role [4]. At present, treatment of OLP is palliative only and topical corticosteroids (TC) are accepted as the first line of treatment [5].

Oral lichen planus can affect any oral site and is present in various forms including: reticular, papular, plaque-like, atrophic, erosive and the rare bullous form [1]. The clinical features alone may be sufficient to make a diagnosis [5, 6].

Oral lichen planus has been associated with other disorders including infection with the hepatitis C virus [1], disturbances in glucose metabolism [7], Sjögren’s syndrome [8], and has been linked to psychological factors [1]. While controversy surrounds the potential for malignant transformation of OLP, currently the World Health Organization (WHO) classifies this disease as a potentially malignant disorder [9-11].

Mouth dryness is one of the most common oral health conditions and has been associated with OLP [8, 10, 12-16]. It may be attributed to xerostomia, salivary gland hypofunction (SGH) or both [17]. Salivary gland hypofunction refers to a diminished salivary flow rate and is based on objective measures of saliva production [18]. Xerostomia is the subjective experience of mouth dryness [18]. The relationship between SGH and xerostomia is a complex one and although the two conditions are related, the mechanisms by which decreases in salivary output are interpreted as the sensation of mouth dryness are not well understood [8]. Unlike the causes of SGH, the causes of xerostomia are poorly established, and have been linked to: medication use [19], impaired oral fluid balance [20], changes in salivary composition [8], inflammatory oral mucosal alterations [8], deficiency states [21], and changes in patient’s perception mechanisms [22].

The literature pertaining to the associations between OLP and xerostomia and/or SGH is limited [8, 10, 12-16]. Some studies have demonstrated a relationship between OLP and SGH [10, 12-16]. In a cross-sectional study, Lundström, *et al*. evaluated salivary flow rate, pH and buffering capacity in 39 OLP subjects [15]. Authors demonstrated that unstimulated salivary flow rate (USFR) was significantly lower in OLP subjects compared to normal reference tables of healthy controls [15]. The reduction in USFR was greater in the OLP subjects, with increasing disease duration [15]. Gandara, *et al*. in two separate studies, examined age-related changes in salivary flow rates, electrolyte and protein concentrations of whole, parotid and labial minor salivary gland saliva in 25 OLP subjects and 25 age and sex matched controls [13, 14]. No significant differences in USFR and stimulated salivary flow rates (SSFR) were noted between the cases and controls [13, 14]. A study by Ramon, *et al*. investigated how different clinical forms of OLP and the extent of oral mucosal involvement affect whole USFR and SSFR in 100 OLP subjects compared to 42 healthy controls [16]. The study found no statistically significant relationships between these variables [16]. Lastly, Bokor-Bratic, *et al*. studied USFRs in 90 OLP subjects and 90 age and sex matched controls and concluded that the mean USFR was within normal range compared to controls [12].

Only two studies investigated the relationship between xerostomia and OLP [8, 10]. Colquhoun and Ferguson examined the relationship between these variables in 77 OLP subjects and 169 age and sex matched controls [8]. A statistically significant association was found between OLP and dry mouth symptoms [8]. Artico, *et al*. examined both xerostomia and salivary gland hypofunction in 38 OLP subjects, 28 subjects with non-OLP mucosal lesions, and 32 healthy controls [10]. Authors concluded that xerostomia and SGH occurred at similar levels within these three groups [10].

To date, to the best of our knowledge, no study exploring the relationship between OLP and mouth dryness has investigated the effect of treatment of OLP, and therefore disease activity, on xerostomia or SGH in those individuals. The aim of this study was therefore to examine the effect of treatment of OLP with TC on xerostomia and SGH in subjects diagnosed with OLP requiring treatment. It is anticipated that results of this study may further improve our understanding of the symptomatology of OLP and some of the mucosal factors contributing to xerostomia.

## MATERIALS AND METHODS

This was an observational before and after comparison study. Participants were selected from a pool of patients attending the oral medicine clinic of the Oral Health Centre of Western Australia (OHCWA), School of Dentistry, University of Western Australia between August 2015 and April 2016.

Subjects included in this study were diagnosed with OLP and presented with symptomatic disease and/or ulcerative/erosive mucosal lesions requiring treatment with TCs. Oral lichen planus subjects who did not speak English; did not consent to participate; did not require treatment with TC, or reported changes in their medical history during the course of the study were excluded.

All subjects were identified following manual screening of OHCWA patient records for the diagnosis of OLP, and all were assessed at the Oral Medicine Clinic between August 2015 and April 2016. At initial presentation and prior to initiating treatment with TC, the age, gender, presence or absence of systemic disease and related medication use were recorded for all participants. All subjects underwent clinical oral examination by an Oral Medicine Specialist, and the presence of OLP lesions was recorded for all intraoral sites. Most cases of OLP [16] were diagnosed on clinical presentation alone (WHO clinical diagnostic criteria) with the remaining cases [3] fulfilling both the WHO clinical and histopathologic criteria [5]. For the purposes of this study, OLP lesions were categorised into two basic types: reticular and ulcerative/erosive. All subjects were asked to complete the 11-question XI [22]. A 10 cm VAS was also used to assess the severity of their xerostomia [23]. Both USFR and SSFR were measured. Salivary gland hypofunction for whole USFR was considered to be ≤ 0.1ml/min [24]. Subjects were requested to refrain from eating or drinking for one hour prior to undertaking the saliva testing. Saliva testing was conducted in three-hour blocks either from 9 am to 12 pm or 1pm to 4pm. Unstimulated saliva was collected by the spitting method [25] over a 10-minutes period. Stimulated saliva was also collected by the spitting method [25]. Subjects were requested to chew on a piece of paraffin wax for five minutes and then spit the saliva into a cup. The collected saliva was weighed in grams and converted to millilitres. The density of saliva is considered to be about 1g/cm^3^ [26]. The pH and buffering capacity of unstimulated saliva was measured using the GC America: Saliva-Check BUFFER^®^ kit (GC America Inc., IL, USA).

Following initiation of treatment with a topical corticosteroid, subjects were reviewed in the Oral Medicine Clinic at time intervals deemed appropriate by the treating consultant. All subjects were followed-up until they became asymptomatic and all ulcerated lesions healed and subjects were no longer using the TC for at least 24 hours to allow for the washout period. At each visit, participants were monitored for changes in their medical conditions and associated medication use.

Statistical analysis was undertaken using the Stata^®^ (version 13.0, Stata Corp LP, College Station, TX, USA) and SAS^®^ (version 9.4, SAS Institute Inc., Cary, NC, USA) software programs. Paired Student’s *t*-test was used to examine associations between OLP and salivary flow rates, pH, buffering capacity, XI and VAS before and after treatment with TC. Multivariate analysis (Generalised linear model in SAS) was used to assess the correlation of results between the xerostomia tools, as well as between the VAS and systemic medications taken by the subjects and between the VAS and the age of subjects. A probability value of less than 0.05 was considered statistically significant in both univariate and multivariate analyses.

Ethics approval was sought and obtained from the Human Research Ethics Committee of the University of Western Australia.

## RESULTS

Fifty-three subjects diagnosed with OLP were recruited for the study with 19 individuals meeting all inclusion criteria during the eight-months data collection period. Reasons for exclusions and characteristics of the 34 excluded individuals are summarised in (Table **1**) . The characteristics of participants are presented in (Table **2**). 

**Fig. (1):**
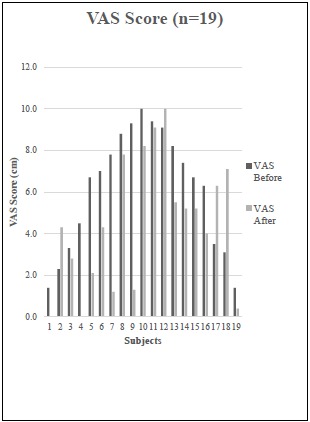
Random effects regression analysis model of correlation of VAS and XI (*P* < 0.001).

**Fig. (2):**
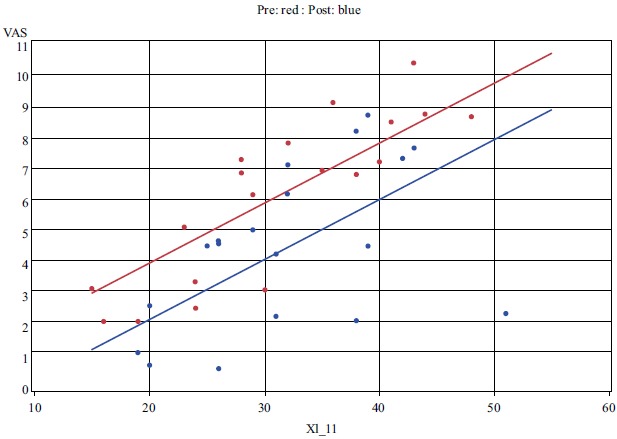
Random effects regression analysis model of correlation of VAS and XI (*P* < 0.001).

All nineteen subjects reported xerostomia before and after treatment with TC. When measuring xerostomia using the VAS tool, there was a statistically significant improvement after treatment with TC (*t*_18_=-2.4, *P =* 0.03) (Fig. **1**). A positive change in xerostomia was experienced by 79% of participants. No statistically significant difference in xerostomia was found using the 11-question XI, although a random effects regression model identified a strong correlation between the XI and the VAS (*P* < 0.001) (Fig. **2**).

When the analysis was restricted to OLP type, a statistically significant reduction in xerostomia was observed on VAS in the subjects with ulcerative OLP (*t*_11_=-2.5, *P =* 0.03) but not for the subjects with the reticular type (*t*_6_=-0.8, *P =* 0.47). No statistically significant relationship was found between medication use and the change in xerostomia based on VAS. Similarly, no significant association was found between age and the change in xerostomia based on VAS.

Salivary gland hypofunction was observed in 47% of OLP subjects before treatment and in 37% of subjects after treatment, and there was no significant change in the USFR *(t*_18_=-0.1, *P* = 0.88*)*. Treatment with TC was not associated with statistically significant differences in stimulated or unstimulated whole salivary flow rates, unstimulated salivary pH or buffering capacity.

## DISCUSSION

The literature pertaining to the associations between mouth dryness and OLP is limited. While these conditions constitute common oral pathoses, both are complex and not entirely understood [8]. Results of studies investigating the relationship between OLP and xerostomia and / or SGH have been conflicting and none of the studies have explicitly taken the effect of treatment of OLP into account [8, 10, 12-16]. Given that inflammatory mucosal changes have been linked to xerostomia [8], the impact of treatment influencing the inflammatory process warrants investigation. To the best of the authors’ knowledge, this is the first study specifically examining the effect of treatment of OLP with TC on xerostomia and SGH.

Nineteen subjects satisfied all inclusion criteria, ranging in age from 53-80 years consistent with previous studies [10, 12, 15, 16]. Women were overrepresented in our study, accounting for 89% of all participants, also in keeping with previous publications [10, 12]. Women have a higher prevalence of OLP [8]; are more likely to seek medical treatment and are therefore more likely to be overrepresented [27]. The female dominance in our study population may also partly explain why all subjects reported xerostomia at presentation. It is well known that the prevalence of SGH and xerostomia is more common in women than men [28]. Some of the reasons for this include: women having smaller salivary glands; producing less saliva; and having lower salivary gland functional capacity than men [28]. In addition to this, women are generally likely to take more medications than their male counterparts [28].

The majority of our participants presented with other co-morbidities (Table **2**) and two of the participants had a concurrent diagnosis of Sjögren’s Syndrome. Furthermore, 63% of the participants took medications which have been associated with mouth dryness. These co-morbidities have also contributed to the high prevalence of xerostomia and SGH in our study population. Nonetheless, none of the participants reported any changes to their medical conditions or any changes to their prescribed medications, including dose changes, over the course of this study.

All subjects reported xerostomia before commencing treatment with TC. When measuring xerostomia using the VAS tool, 79% reported a statistically significant improvement after treatment with TC. No statistically significant difference in xerostomia was found using the XI, although a correlation was observed between the two xerostomia tools. A correlation between VAS and Likert scale based assessments of xerostomia has been previously demonstrated [29]. Lack of a statistically significant difference in xerostomia using the XI in this study may be due to a number of factors, including our small sample size. Additionally, the XI has a tendency to assess xerostomia over time while VAS assesses the condition at a specific point in time [30], which arguably may be more applicable in a study of a dynamic disease such as OLP, where the specific aim is to establish the effect of treatment at a particular point in time [31]. Furthermore, the XI includes questions relating to extra-oral symptoms, which were not the focus of our study [32] and the answer options may have been confusing to some participants [22]. Finally, Likert scales, of which the XI is an example, have a tendency for scores towards the midline instead of scores at the extremes, whereas VAS is a continuous scale and is more sensitive at detecting small differences [33].

Xerostomia is a complex, multifactorial and, overall, a poorly understood phenomenon [20]. While xerostomia has been linked to mucosal diseases such as OLP, the exact mechanisms have not been explained. It has been proposed that the inflamed mucosa in OLP subjects may be associated with altered surface texture, contributing to the sensation of mouth dryness [8]. How altered surface texture may contribute to xerostomia has not been clarified [8]. Results of our study seem to support a role for inflammatory mucosal injury (as occurs in OLP) in xerostomia, as suppression of inflammation with TC has been associated with improvement in dry mouth symptoms, in the absence of any significant changes in salivary flow rates. This is further supported by the fact that the most significant improvement in xerostomia occurred in individuals with the more severe disease, namely with erosive/ulcerative lesions, as compared to individuals with reticular disease only.

Impaired oral fluid balance has been linked to xerostomia and may be impacted by mucosal disease [20]. Saliva forms a thin film that covers the oral cavity, varying in thickness from about 72-100 microns, depending on the site [20]. This steady state volume of residual fluid reflects a balance between fluid secretion by the salivary glands and fluid loss, as can occur through swallowing, absorption across the mucosal epithelium and evaporation [34]. It has been shown that individuals with xerostomia, who do not necessarily present with SGH, exhibit abnormally low salivary film thicknesses [20]. Chronic mucosal inflammation, as occurs in OLP, may disturb this oral fluid balance by compromising the integrity and function of the oral epithelium, specifically, by adversely affecting the normal fluid and electrolyte absorption, and secretion by the epithelium, a dysfunction which has been attributed to xerostomia [34-36]. Loss of epithelium, as occurs in ulceration, also likely affects the formation of oral mucosal pellicle, which lubricates the oral soft tissues [34]. Heat associated with an inflammatory reaction may enhance surface fluid evaporation.

While the exact mechanisms require investigation, results of this study support a link between OLP and xerostomia and suggest that the suppression of the inflammatory reaction, the healing of mucosal lesions and restoration of mucosal integrity following the use of TC contributed to the improvement in the severity of xerostomia in the OLP subjects studied. Control of OLP with TC was not associated with complete resolution of xerostomia and this was not expected due to the comorbidities of the study population and the associated high prevalence of SGH. Results of this study also support earlier observations that xerostomia may form part of the symptomatology of OLP in some individuals.

Treatment with TC was not associated with statistically significant differences in stimulated or unstimulated whole salivary flow rates, unstimulated salivary pH or buffering capacity. This again was not unexpected. OLP is not primarily a disease of the salivary glands [37] and therefore treatment with TC was not expected to significantly influence salivary gland function nor the salivary pH or the buffering capacity, both of which are flow rate dependent [38]. While it was not possible to collect all salivary samples at the same time of day to compensate for the potential influence of the circadian rhythm, Osalin, *et al*. have questioned the significance of the circadian rhythm on USFR, suggesting that USFR can be measured during normal clinic hours [39].

This study has limitations including a small sample size due to limited availability of patients and absence of histological confirmation of diagnosis in some cases. Nonetheless, all participants presented with clinically obvious diagnosis [6], as was determined by an Oral Medicine Specialist, and all subjects responded to treatment with TC, as was expected of the condition. The value of histological confirmation of the classic reticular form of OLP has also been previously questioned [6]. Future studies examining the relationship between OLP and mouth dryness should consider the potential impact of OLP treatment and disease activity and further explore the mechanism underpinning xerostomia in OLP subjects.

## CONCLUSION

To the best of the authors’ knowledge, this is the first study examining the effect of treatment of OLP with TC on xerostomia and SGH. Within the limitations of this study, results obtained suggest that treatment of OLP with TC may decrease the severity of dry mouth symptoms and that xerostomia may form part of the symptomatology of OLP in some individuals.

## Figures and Tables

**Table 1:** Characteristics of excluded individuals and reasons for exclusions.

		Number of Exclusions	
		Male	Female
Total individuals excluded		7	27
		Mean age: 79y Age range:57-76y	Mean age: 71y Age range: 46-89y
Reason:	Withdrew consent to participate	1	2
	Participant not presenting in the right OLP state at follow-up during the course of study (*e.g*. persistent mucosal ulceration; persistent symptoms; continued corticosteroid use)	5	22
	Change in diagnosis of oral mucosal disease during the course of study	1	3

**Table 2:** Characteristics of participants included in study.

		Number of Participants	
		Male	Female
Total individuals included		2	17
		Mean age: 67y Age range: 63-71y	Mean age: 73y Age range: 53-80y
OLP type	Reticular	0	7
	Ulcerative / Erosive	2	10
Participants taking medications		2	16
Participants taking medications affecting salivary flow		2	10
Medical conditions of participants	Haematological disease	0	2
	Cardiovascular disease	2	10
	Respiratory disease	1	3
	Endocrine and metabolic	1	5
	Gastrointestinal disorders	2	6
	Genitourinary disease	0	1
	Musculoskeletal disease	1	9
	Connective tissue disease	0	2
	Mental and behavioural disorders	0	2
	Skin Disease	0	3
	Diseases of the nervous system	1	5
	Ocular Disease	0	4
	Neoplasms	0	4
